# Prevalence of Pathogenic Variants and Eligibility Criteria for Genetic Testing in Patients Who Visit a Memory Clinic

**DOI:** 10.1212/WNL.0000000000210273

**Published:** 2025-01-27

**Authors:** Sven J. Van Der Lee, Marc Hulsman, Rosalina Van Spaendonk, Jetske Van Der Schaar, Janna Dijkstra, Niccoló Tesi, Fred van Ruissen, Mariet Elting, Marcel Reinders, Itziar De Rojas, Corien C. Verschuuren-Bemelmans, Wiesje M. Van Der Flier, Mieke M. van Haelst, Christa de Geus, Yolande Pijnenburg, Henne Holstege

**Affiliations:** 1 Genomics of Neurodegenerative Diseases and Aging, Human Genetics, Vrije Universiteit Amsterdam, Amsterdam UMC location VUmc, the Netherlands;; 2 Alzheimer Center Amsterdam, Neurology, Vrije Universiteit Amsterdam, Amsterdam UMC location VUmc, the Netherlands;; 3 Amsterdam Neuroscience, Neurodegeneration, the Netherlands;; 4 Department of Human Genetics, Amsterdam Reproduction and Development Research Institute, Amsterdam UMC, University of Amsterdam, the Netherlands;; 5 Clinical Genetics, Dept. Human Genetics, Amsterdam UMC, the Netherlands;; 6 Delft Bioinformatics Lab, Delft University of Technology, Delft, the Netherlands;; 7 Research Center and Memory Clinic, Ace Alzheimer Center Barcelona, Universitat Internacional de Catalunya, Barcelona, Spain;; 8 CIBERNED, Network Center for Biomedical Research in Neurodegenerative Diseases, National Institute of Health Carlos III, Madrid, Spain;; 9 Department of Genetics, University of Groningen, University Medical Center Groningen, the Netherlands;; 10 Epidemiology and Data Science, Vrije Universiteit Amsterdam, Amsterdam UMC location VUmc, the Netherlands;; 11 Amsterdam Reproduction and Development, Amsterdam UMC, the Netherlands; and; 12 Emma Center for Personalized Medicine, Amsterdam UMC, the Netherlands.

## Abstract

**Background and Objectives:**

Identifying genetic causes of dementia in patients visiting memory clinics is important for patient care and family planning. Traditional clinical selection criteria for genetic testing may miss carriers of pathogenic variants in dementia-related genes. This study aimed identify how many carriers we are missing and to optimize criteria for selecting patients for genetic counseling in memory clinics.

**Methods:**

In this clinical cohort study, we retrospectively genetically tested patients during 2.5 years (2010–2012) visiting the Alzheimer Center Amsterdam, a specialized memory clinic. Genetic tests consisted of a 54-gene dementia panel, focusing on Class IV/V variants per American College of Medical Genetics and Genomics guidelines, including *APP* duplications and the *C9ORF72* repeat expansion. We determined the prevalence of pathogenic variants and propose new eligibility criteria for genetic testing in memory clinics. The eligibility criteria were prospectively applied for 1 year (2021–2022), and results were compared with the retrospective cohort.

**Results:**

Genetic tests were retrospectively performed in in 1,022 of 1,138 patients (90%) who consecutively visited the memory clinic. Among these, 1,022 patients analyzed (mean age 62.1 ± 8.9 years; 40.4% were female), 34 pathogenic variant carriers were identified (3.3%), with 24 being symptomatic. Previous clinical criteria would have identified only 15 carriers (44% of all carriers, 65% of symptomatic carriers). The proposed criteria increased identification to 22 carriers (62.5% of all carriers, 91% of symptomatic carriers). In the prospective cohort, 148 (28.7%) of 515 patients were eligible for testing under the new criteria. Of the 90 eligible patients who consented to testing, 13 pathogenic carriers were identified, representing a 73% increase compared with the previous criteria.

**Discussion:**

We found that patients who visit a memory clinic and carry a pathogenic genetic variant are often not eligible for genetic testing. The proposed new criteria improve the identification of patients with a genetic cause for their cognitive complaints. In systems without practical or financial barriers to genetic testing, the new criteria can enhance personalized care. In other countries where the health care systems differs and in other genetic ancestry groups, the performance of the criteria may be different.

## Introduction

Dementia affects millions worldwide and is commonly a complex interplay of multiple genetic and environmental factors, a minority of patients has a familial (also called monogenic) form of dementia.^1-3^ Identifying these familial dementia cases and their underlying pathogenic genetic variants (PGVs) is crucial because it can guide diagnosis and enables presymptomatic testing for relatives.^4-6^ Advances in next-generation sequencing (NGS) have identified over 50 genes associated with familial dementia.^7,8^ PGV identification also supports enrolment in clinical trials and participation in research, such as the Dominantly Inherited Alzheimer Network Trial Unit^9^ for Alzheimer disease (AD)^10^ and the Genetic Frontotemporal dementia Initiative^11^ for frontotemporal dementia (FTD).

However, despite increasing interest in genetic testing^12^ and decreasing NGS costs,^13^ its use in general memory clinics remains limited, mostly reserved for early-onset dementia clinics.^2,3^ For example, at the Amsterdam Alzheimer Center, approximately 10%–15% of patients undergo testing, with PGVs detected in roughly 10% of tested cases.^1^ Studies suggest that broader testing could reveal PGVs in up to 12.6% of young-onset dementia cases.^2^ Nevertheless, many PGV carriers remain undiagnosed due to stringent eligibility guidelines that often require early onset, clinical diagnosis, and positive family history.^7,13-16^ The criteria miss carriers, especially in cases of misdiagnosis, variable onset, or complex family histories. For example, as with *C9ORF72* repeat expansion carriers^17^ that manifest variably as clinical FTD,^10^ amyotrophic lateral sclerosis (ALS),^17,18^ psychiatric disorders,^19^ or other syndromes.^20,21^ In addition, the occurrence of “de novo” mutations complicates reliance on family history alone for diagnosis.^10^

In summary, despite the important implications and increasing patient interest, it is likely that many PGV carriers are not offered genetic testing and thus remain unidentified. For patients living in countries without practical or financial issues concerning genetic testing, the optimization of the eligibility criteria for offering genetic testing could improve detection of PGV carriers in dementia care.

## Methods

In this article, we describe 2 complementary studies embedded in the Alzheimer Center Amsterdam (eFigure 1). First, we investigated the prevalence of PGVs in a retrospective cohort including all patients who visited the memory clinic in a period of time. This allowed us to formulate novel criteria to select patients eligible for genetic testing. Second, we validated these criteria by applying the criteria to all patients who visited the clinic over a 1-year period (prospective study cohort).

### Patient Population

The Alzheimer Center Amsterdam is a specialized memory clinic for young-onset dementia. The clinic is a so-called tertiary referral clinic that receives referrals from specialists from other memory clinics—for example, for a second opinion—but also direct referrals from general practitioners. A general practitioner can refer if there are cognitive complaints that warrant investigation by a specialist. Specialist referrals are second opinions, cases where the specialist has diagnostic doubts or by wish of the patient to participate in research. All patients undergo the same diagnostic trajectory which did not significantly change between 2010 and 2022.^22,23^ This includes a medical and neurologic investigation, blood tests, EEG, neuropsychological assessment, brain magnetic resonance imaging, and optional CSF analysis.^22,23^

### Standard Protocol Approvals, Registrations, and Patient Consents

The vast majority of patients that visit the Amsterdam Alzheimer Center consent to use their medical data for scientific research and are included the Amsterdam Dementia Cohort.^22,23^ The consent for storage of their DNA in a dedicated biobank is optional.^22^ The Amsterdam Dementia Cohort, storage and sequencing of DNA, has been approved by the Medical Research Ethics Committee of Amsterdam UMC, location VUMC (2016.061, 2017.315). In the retrospective study, genetic analysis was done under scientific research consent, such that patients did not receive the outcomes of the genetic analysis. In the prospective study, genetic testing was done after consent of the patient as part of clinical care, such that outcomes of the genetic analysis were shared with patients.

### Study Cohorts

#### Retrospective Cohort Study

The retrospective cohort included patients who consecutively visited the clinic between January 1, 2010, and June 30, 2012, and who consented to use their medical data. Patients were excluded if they did not give consent for DNA research, if there was no DNA available or if sequencing failed because of poor DNA or sequencing quality. In the clinical records of all patients, we checked whether clinicians found a pathogenic variant up until the end of 2022, that is, at least 10 years after initial presentation, and we evaluated whether relevant clinical changes were reported for all identified PGV carriers in this study.

#### Prospective Validation Cohort

**Figure 1:**
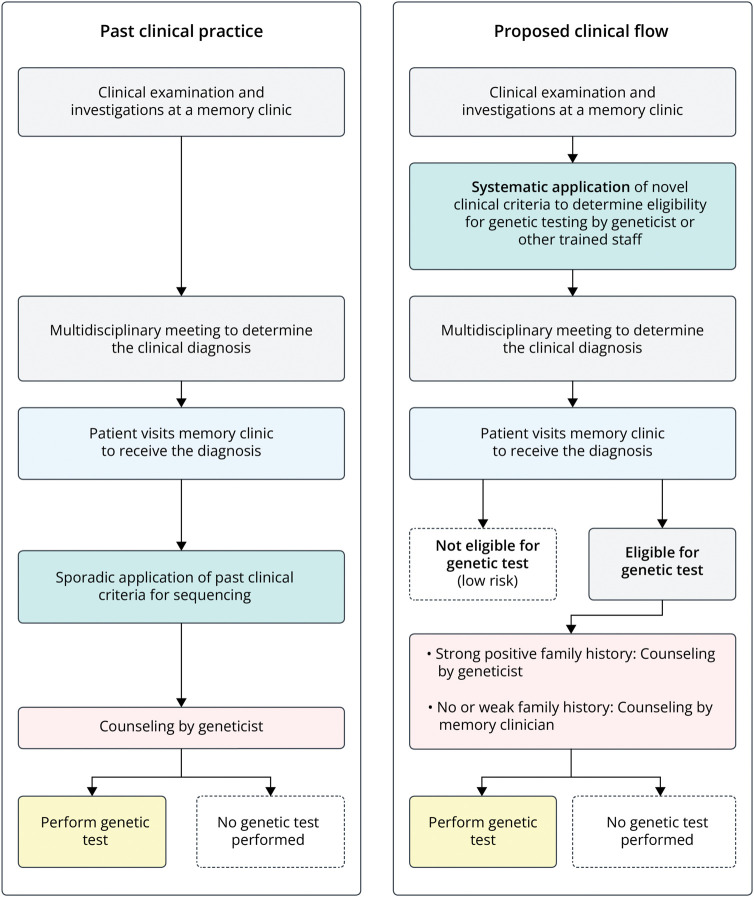
Old and Newly Proposed Clinical Practice Around Genetic Testing in Memory Clinics The left block shows the clinical practice in most memory clinics. Eligibility criteria for genetic testing (if present) are sporadically applied to patients who visit a memory clinic. Based on current finding, we propose a different clinical workflow. In this workflow, the clinician assesses the eligibility using the proposed criteria for genetic testing for all patients at time of the initial diagnosis in the multidisciplinary meeting that precedes diagnosis in most memory clinics. Based on this assessment, the patients are eligible or not. Only patients with a strong positive family history were referred to a clinical geneticist. If this strong suspicion was not present, the neurologist could counsel and, if indicated, order the test directly or refrain from testing.

In the prospective cohort, we included patients who visited the Alzheimer Center Amsterdam between September 2021 and September 2022 and provided informed consent for research. The clinical practice was adapted as shown in Figure 1. We now applied the eligibility criteria for genetic testing systematically to *all* patients at time of diagnosis. Four months after inclusion of the last patient, at the end of 2022, we checked all medical records and recorded if counseling by a clinical geneticist had taken place, if a genetic test was performed. If a test was performed, we recorded if a PGV and variants of unknown significance (VUS) was reported (Class III variants according to the American College of Medical Genetics and Genomics [ACMG]^3^). VUS are reported only if the gene in which they are found matches with the phenotype of the patient.

### Genetic Tests

We tested for PGVs in a dementia gene panel (targeted exome-sequencing) consisting of 54 genes (eTable 1), *APP* duplications, and the *C9ORF72* hexanucleotide repeat expansion. Sex was determined by self-identification and matched the biological sex in the genetic data. The panel of genes was determined by clinical laboratory specialist and a clinical geneticist by review of literature in 2016. PGVs are defined as Class IV and V based on the ACMG.^3^ Variants of Class V, so called “pathogenic variants,” are variants that were previously observed to segregate with disease and/or with an established biological pathologic effect. Variant classified as Class IV, so called “likely pathogenic variants,” are variants with genetic characteristics of Class V variants but have not been observed previously. The genetic tests performed in the retrospective cohort were nearly identical to the genetic tests performed in the prospective validation cohort, as part of routine clinical procedures (in 2021). Description of genetic testing is described in the eMethods. Clinical phenotype sometimes warranted a targeted analysis of variants in other genes than those in the panel. These results were extracted from medical records. S.v.d.L. and clinical laboratory specialist R.V. were involved in evaluation of genetic variants for pathogenicity in the research-based retrospective study, while the variants in the prospective study were judged as part of clinical procedures by R.V. This evaluation included variant frequency in public (such as the Genome Aggregation Database^24^) and previous evaluations of variants observed in Dutch patients (accessible for clinical laboratory specialists).

### Criteria to Determine Eligibility for Genetic Testing

#### Criteria Applied (From 2017) Until 2021

eTable 2 shows the previous criteria applied to determine eligibility for genetic testing at the Alzheimer Center Amsterdam.^1^ We evaluated the old eligibility criteria in all retrospectively because genetic testing in the period of the retrospective study (2010–2012) was performed sporadically and gene-by-gene.^25^

#### Novel Criteria to Determine Eligibility for Genetic Testing

The proposed steps in the new criteria were based on the clinical diagnoses (cognitively normal, mild cognitive impairment [MCI], AD, FTD, etc). Within the clinical diagnosis, we calculated the prevalence of PGV and separated the prevalences based on age at presentation and family history. If there were differences in prevalence of PGVs observed in the clinical diagnostic groups, by age at presentation and/or family history a substep was included after the clinical diagnosis in the criteria. Detailed description of the analysis of the retrospective cohort is in the eMethods.

#### Prospective Application of New Eligibility Criteria

Then, we prospectively applied the eligibility criteria to all new patients in the Alzheimer Center Amsterdam for 1 year. We report the number of eligible patients, number of patients tested, and PGV detection after 1 year of implementation. In practice, patients’ diagnosis and family history were discussed before the multidisciplinary meeting by a research physician (S.v.d.L.) and clinical geneticist (C.G., M.W.E.). They decided whether there was an indication for genetic testing, and, if so, whether there was a high suspicion of finding a PGV based on family history. At the multidisciplinary meeting of the memory clinic, this outcome would be shared, and if there was high suspicion, a referral for counseling would be recommended to the treating neurologists. In some instances, the advice was modified based on new insights revealed at the multidisciplinary meeting. The neurologist then offered genetic testing or counseling to the patient at the time the diagnosis was communicated to the patient. In this consultation with the neurologist, patients decided either not to opt for genetic testing, opt for genetic testing, or to be referred to a clinical geneticist for counseling. In some instances, the neurologist postponed the decision to a follow-up visit. Four months after inclusion of the last patient, at the end of 2022, we checked all medical records and recorded if counseling by a clinical geneticist had taken place, if a genetic test was done, if a PGV or VUS was identified (Class III variants according to the ACMG^3^).

### Data Availability

Anonymized individual deidentified participant data and genetic data not published within this article will be made available by on reasonable request from any qualified investigator by contacting the corresponding author and prof. W.M. van der Flier.

## Results

**Figure 2:**
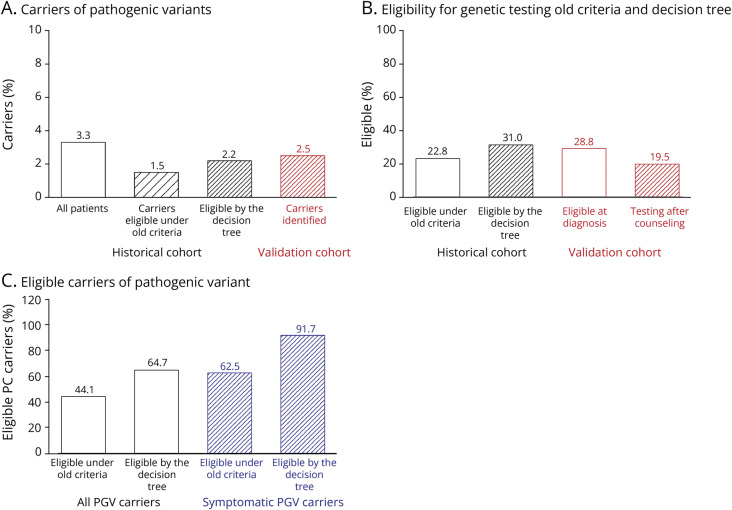
Main Results of the Analysis of the Retrospective and Prospective Cohort Patients are eligible for testing in retrospective and prospective cohort (A), percentage of PGV carriers of all patients in retrospective and prospective cohort (B), number of PGV carriers that were eligible for genetic testing in retrospective cohort. PGV = pathogenic genetic variant.

The retrospective cohort included 1,138 patients who consecutively visited the clinic between 2.5 years. We excluded 116 patients; 45 patients did not give consent for DNA research (4.1%), for 60 patients, no DNA was available (5.5%), and for 11 patients, DNA or sequencing quality was poor (1.0%). Finally, we performed a genetic test retrospectively in 1,022 (90%) of the patients. Of the 1,022 analyzed patients, 40.4% were female and the mean age at presentation was 62.1 years (±8.9). The most frequent diagnoses observed were 309 patients with AD (30.2%), 225 (22%) with subjective complaints, 149 (14.5%) with MCI, and 104 (10.2%) with a primary psychiatric diagnosis (Table 1). Demographics of patients who were genetically tested were similar to those who were not genetically tested (eTable 3). Using the genetic tests, we identified 34 PGV carriers among 1,022 tested patients (3.3% prevalence) (Figure 2A). The most frequently observed PGV was the *C9ORF72* hexanucleotide repeat expansion (N = 8, 23.5% of all PGVs). The second most frequently affected gene was *PSEN1* (NP_000012.1, N = 5, p.Ala79Val, 15% of all PGVs), followed by variants in *NOTCH3* (NP_000426.2, N = 3, p.Arg1231Cys, p.Arg578Cys, p.Arg640Cys), *SORL1* (NP_003096.1, c.401_402+2delATGT, p.His962Profs*45, p.Pro712Leufs*54), and *TARDBP* (NP_031401.1, p.Ala382Thr[1], p.Asn267Ser[2]). We assumed that patients with the same PGV share a common ancestor with the founder variant; however, we could not confirm relatedness based on identity by descent analyses or by connecting pedigrees. Two patients had a mutation detected in clinic outside the gene panel; a deletion in *SPG4* in a patient with cognitive decline, slowly progressive muscle weakness and spasticity, and a 44 CAG-repeat in the Huntington (*HTT*) gene (>40 repeats is pathogenic) in a patient with dementia and subtle chorea. All genes with PGVs are shown in eTable 4. For privacy reasons, individual patient characteristics and family history of patients with a PGV are not reported.

**Table 1:** Comparing Gene Carriers With Noncarriers

Group	All (N = 1,022)	A PGV present (N = 34)	No PGV (N = 988)	*p* Value	Statistic
N-female participants (%)	413 (40.4)	17 (50)	396 (40.1)	0.327	0.96
Age (y)	62.1 (8.9)	58.9 (9.3)	62.2 (8.9)	0.056	1.97 (*t*)
Education^a^ (low/intermediate/high) (%)	4/58/38	0/59/41	4/58/38	0.473	1.49
Diagnosis					
Subjective complaints (%)	225 (22)	3 (8.8)	222 (22.5)	0.093	2.81
MCI (%)	149 (14.6)	4 (11.8)	145 (14.7)	0.821	0.05
PPD (%)	104 (10.2)	4 (11.8)	100 (10.1)	0.982	0.00
AD (%)	309 (30.2)	8 (23.5)	301 (30.5)	0.499	0.45
FTD (%)	51 (5)	6 (17.6)	45 (4.6)	2.3E-03	9.28
DLB (%)	35 (3.4)	0 (0)	35 (3.5)	0.524	0.40
PPA (%)	3 (0.3)	0 (0)	3 (0.3)	1.000	0
Pure vascular dementia (%)	17 (1.7)	2 (5.9)	15 (1.5)	0.203	1.62
Other dementias (%)	30 (2.9)	1 (2.9)	29 (2.9)	1.000	0
Other neurology (%)	33 (3.2)	1 (2.9)	32 (3.2)	1.000	0
Unclear diagnosis (%)	66 (6.5)	5 (14.7)	61 (6.2)	0.102	2.67

Abbreviations: AD = Alzheimer disease; DLB = dementia with Lewy bodies; FTD = frontotemporal dementia; MCI = mild cognitive impairment; PPA = primary progressive aphasia; PPD = primary psychiatric diagnosis.

Categorical variables were compared using a χ^2^ test. Monte Carlo simulation or continuity correction was used if one of the cells in the tables was less than 5. For age, an independent *t* test was used (*t*).

a Education levels defined based on the International Standard Classification of Education 2011.

The demographic characteristics of PGV carriers were similar to those not carrying a PGV (i.e., noncarriers) (Table 1). The median age at presentation of the 34 PGV carriers was 58 years (interquartile range [IQR] 52–67) and 62 years (IQR 56–69) for 988 noncarriers (*p* = 0.056, *t* test). More specifically, the age at presentation was younger than 60 years for 16 PGV carriers (47%), between 60 and 70 years for 13 PGV carriers (38%), and older than 70 years for 4 PGV carriers (11%). Of the 34 PGV carriers, 17 were diagnosed with dementia (50%), 3 with subjective memory/cognitive complaints (8.8%), 4 with MCI (11.8%), 4 with a primary psychiatric diagnosis (11.8%), and 5 had an unclear diagnosis of dementia (14.7%). Compared with noncarriers, only FTD was overrepresented in PGV carriers (17.6% vs 4.6%, χ^2^ = 9.2, *p* = 2.3 × 10^−3^). Of the PGV carriers, 50% reported having a first-degree relative with dementia (any dementia in siblings or parents), which was similar to 49% of the noncarriers (χ^2^ = 0, *p* = 1).

### Eligibility Criteria for Genetic Testing in Dementia Patients

**Figure 3:**
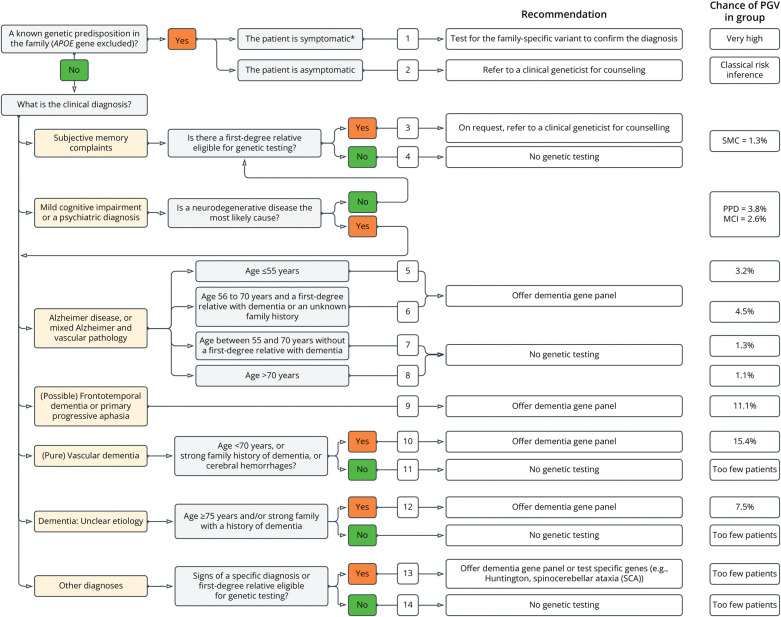
Proposed Criteria to Determine Eligibility for Genetic Testing in a Memory Clinic Setting The dementia gene panel consists of 54 dementia related genes, the *APP* duplication, and the *C9ORF72* repeat expansion. First-degree relatives are parents, siblings, and children. *Means symptomatic as determined by a neuropsychological investigation. The outcomes of the criteria are numbered and correspond to the rows of Table 2. The chance to find a pathogenic variant based on the retrospective cohort is shown on the right of the figure. In addition to family history of dementia a family history of severe late-onset psychiatry, amyotrophic lateral sclerosis and Parkinson disease dementia should be considered as well. MCI = mild cognitive impairment; PPA = primary progressive aphasia; PPD = primary psychiatric diagnosis.

We propose new eligibility criteria to determine eligibility for genetic testing, shown in Figure 3. The steps in eligibility criteria are based on the clinical diagnoses (cognitively normal, MCI, AD, FTD, etc.) a patient in a memory clinic can get. Within the clinical diagnosis, we calculated the prevalence of PGV and separated the prevalences based on age at presentation and family history. If there were differences in prevalence of PGVs observed in the clinical diagnostic groups, by age at presentation and/or family history, a substep was included after the clinical diagnosis in the eligibility criteria.

#### Retrospective Application of Old and Revised Criteria

In total, 533 patients visited the Alzheimer Center Amsterdam between September 2021 and September 2022, of which 97% (517) gave informed consent to use their medical data for research. The eligibility criteria for genetic testing were systematically applied in *all* patients at time of diagnosis. In total, 515 patients were included in this validation cohort.

Based on clinical information, the eligibility criteria used from 2017 until 2021 (eTable 1) were applied retrospectively to all patients to determine if patients would have been eligible for genetic testing. This allowed us to compare the identification of PGV carriers between the old and the new criteria (Figure 2). Based on the old eligibility criteria, we estimate that 261 of 1,022 patients would have been eligible for genetic testing (22.8%) (Figure 2B), allowing the identification of only 15 PGV carriers (44.1%), whilst the other 19 PGV carriers would have been missed (56%) (Figure 2C). Based on the new formulated eligibility criteria, we estimate that 317 (31.0%) of 1,022 patients would have been eligible for testing (Figure 2B), which would have led to the identification of 22 out of 34 PGV carriers. This is 2.2% of the retrospective cohort (Figure 2C). We then assessed how many symptomatic PGV carriers would have been eligible in the old and revised criteria. Symptomatic patients included patients with MCI and amyloid biomarkers, patients with dementia due to AD, FTD, dementia with Lewy bodies, vascular dementia, other rare dementias/neurologic symptoms, and those with an unclear etiology. A total sample of 606 (59%) was symptomatic. In this sample, 24 individuals carry a PGV (24/606 = 3.9%). Under the old criteria, 15 patients (15/24 = 62.5%) would have been eligible for genetic testing, whereas with the new criteria, 22 patients would have been eligible (22/24 = 91.7%). The results of eligibility and percentage of PGV carriers the retrospective cohort split by diagnostic group (eFigure 2) and by age (eFigure 3) is in the supplements.

#### Prospective Application of the Revised Criteria in a Memory Clinic

**Figure 4:**
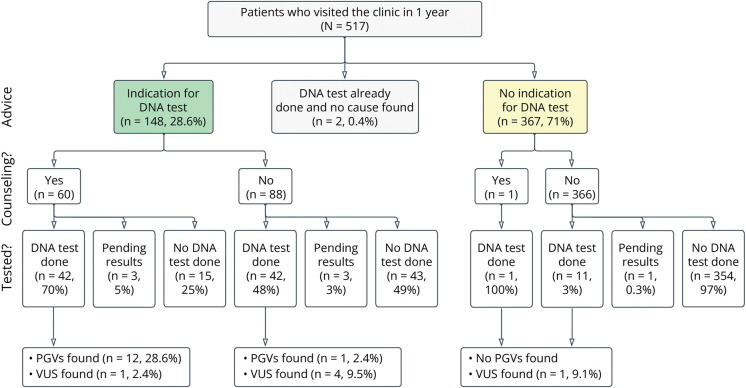
Results of 1-Year Prospective Implementation in Clinical Care Green = those eligible for genetic testing. Yellow = those not eligible for genetic testing. PGV = pathogenic genetic variant; VUS = variant of unknown significance.

We included 517 of patients in the prospective cohort (Figure 4 and Table 2), and 16 patients did not provide consent to use their medical data for research (97% participation). The average age at presentation was 64.1 years (IQR 59–70), and 41.6% of patients were female. Two patients had already undergone genetic testing prior to their visit (2/517 = 0.4%). Of the remaining 515 patients, a total of 148 patients (28.6%) were eligible for genetic testing at time of diagnosis. This was comparable with the estimation based on the retrospective cohort (31%). Four months after the last patient was included in the prospective cohort, 103 patients had consented to genetic testing (103/517 = 20%) of whom, at the multidisciplinary meeting, 90 were considered eligible according to the new criteria (90/103 = 88%). In 13 patients, who were not eligible for genetic testing by the criteria at the first visit, the neurologist decided to perform genetic testing at a later visit and no PGVs were found (reasons not recorded). Of the 90 patients who were eligible for genetic testing, 13 carried a PGV (diagnostic yield: 14.4%), in accordance with a 2.5% prevalence of genetic dementia in the total cohort (13/515, Figure 2A). PGVs were observed in *PSEN1* (1x), *PSEN2* (2x), *NOTCH3* (3x), *MAPT* (1x), *HTRA1*(1x), *CTSF* (2x compound heterozygote), *AMACR* (1x compound heterozygote, specific test), *AARS2* (2x compound heterozygote), and a duplication in the region 16q24.2q24.3 (specific test performed because of concomitant intellectual disability) (all variants are in eTable 5). It is noteworthy that we did not identify any carriers of the FTD/ALS associated *C9ORF72* repeat expansion or *SORL1* variant carriers. The results of eligibility and percentage of PGV carriers the prospective cohort split by diagnostic group (eFigure 2) and by age (eFigure 3) is in the supplements.

**Table 2:** Results of the Validation Cohort

Flowchart outcome		Advice in eligibility criteria	N-patients (% of total)	N-eligible (% of group)	N-tested (% of eligible)	N-carrier (% of tested)
1	PGV known: symptomatic	Test for PGV	1 (0.2)	1 (100)	1 (100)	1 (100)
2	PGV known: asymptomatic	Refer	2 (0.4)	2 (100)	1 (50)	1 (100)
3	SCD/psychiatry/MCI: eligible	Refer	5 (1)	5 (100)	3 (60)	2 (66.7)
4	SCD/psychiatry/MCI: not eligible	No test	169 (32.7)	0	0	0
5	AD: younger than 55 y	Offer test	16 (3.1)	16 (100)	5 (31.2)	0
6	AD: 55–70 y with 1e degree relative with dementia	Offer test	52 (10.1)	52 (100)	23 (44.2)	0
7	AD: 55–70 y test no 1e degree relative with dementia	No test	59 (11.4)	3 (5.1)	6 (200)^a^	0
8	AD: older than 70 y: test not indicated	No test	61 (11.8)	0	2	0
9	FTD/PPA: test indicated	Offer test	21 (4.1)	20 (95.2)	16 (80)	1 (6.2)
10	Vascular dementia: younger than 70 or family history or cerebral haemorrhage	Offer test	7 (1.4)	7 (100)	4 (57.1)	3 (75)
11	Vascular dementia: other	No test	2 (0.4)	0 (0)	0	0
12	Unclear etiology (<75 y)	Offer test	35 (6.8)	34 (97.1)	27 (79.4)	3 (11.1)
13	Other diagnoses: signs of specific cause or 1e degree relative fulfilling eligibility criteria	Offer test or test specific genes	7 (1.4)	7 (100)	6 (85.7)	2 (33.3)
14	Other diagnoses: test not indicated	No test	49 (9.5)	1 (2)	3 (300)^a^	0 (0)

Abbreviations: AD = Alzheimer disease; FTD = frontotemporal dementia; PPA = primary progressive aphasia.

The outcomes of the new eligibility criteria in Figure 3 are numbered and correspond to the rows of table. N-eligible is the number of patients determined eligible for genetic testing.

a The percentage of patients tested is higher than the number of patients eligible because neurologists performed DNA tests in patients not eligible. Possibly because of changes in symptoms, family history, diagnosis, or on patients' request.

#### Comparison of Expected and Observed Number of PGV Carriers

According to the “old” criteria, 3.33% of the patients in the retrospective cohort carried a PGV and of these 1.46% of patients would be expected to be found with the old criteria (Figure 2). In the prospective cohort of 515 novel patients, these percentages would translate to 17 patients with a PGV, and we estimate that with the old criteria, 7.5 patients would be identified. In reality, we identified 13 PGV using the new criteria. This is 76% (13/17) of the expected PGV carriers in the prospective cohort and 73% more than expected PGV carriers (13/7.5 × 100 − 100% = 73%).

#### Variants of Unknown Significance

We observed a VUS in 6 out of 103 tested patients (5.8%). In families of VUS carriers, no additional segregation analysis or functional analysis could be performed in family members because there were no alive family members with disease. We recommended evaluation of pathogenicity of these genetic variants after 5–10 years or as soon as family members develop cognitive complaints.

### Implementation of Counseling in the Prospective Cohort

In the validation cohort, 60 patients of those eligible for testing were counselled by a clinical geneticist (40.5%) and 88 patients were counselled by a neurologist (59.5%). After counseling by a clinical geneticist, 45 (75%) agreed to proceed with testing, while 15 patients decided against testing (25%). After counseling by the neurologist 45 (51%) proceeded with testing, while 43 patients decided against testing (49%). Of the 42 patients who had completed a genetic test after counseling by the clinical geneticist, 26.6% (12/42) carried a PGV. In contrast, of the 42 patients that completed a genetic test after counseling by the neurologist 2.4% (1/42) carried a PGV. The higher percentage of PGV carriers in the group counselled by a clinical geneticist confirms patients with a very suspect family history should be referred.

## Discussion

Identification of PGVs can provide vital information to patients with dementia and their family members. Here, we presented novel criteria that can aid clinicians in memory clinics to select patients for genetic testing. We found that, based on the criteria used until 2021, only 65% of all symptomatic patients with a PGV would be eligible for genetic testing and this increased to 91% with the new criteria. To validate the eligibility criteria, we prospectively assessed all patients for 1 year and found that we identified 73% more PGV carriers. Hence, offering genetic tests to the right patients visiting specialized memory clinics can lead to improved identification of PGVs.

This study has several strengths. First, embedded in the Alzheimer Center Amsterdam, the study ensured a uniform and streamlined workflow for all patients.^22^ This consistency and accuracy in the clinical assessments improved reliability of reported numbers of expected patients with PGV by diagnosis. Second, approximately 95% of all patients entering the clinic participate in scientific research, which minimizes inclusion bias related to age and diagnosis. The study also has challenges. It is important to note that we used a single site, the Alzheimer Center Amsterdam, that specializes in young-onset dementia with many patients asking for a second opinion. This likely led to a higher percentage of eligibility and a larger number of PGV carriers compared with an average memory clinic. While this was crucial for constructing and validating the new criteria, it also limited the number of patients older than 75 years. External studies have shown lower numbers of patients with deleterious variants in this age group.^2^ Further validation in less specialized clinics with older patients, and fewer additional diagnostic tests is warranted. An additional challenge in genetic testing is the identification of gene VUS. We did not systematically assess the number of VUS (Class 3 variants) in our retrospective cohort. Still, in the validation cohort, the number of observed VUS was low (5.8%) and had no clinical consequences. Despite the study's use of a panel of 54 genes, we acknowledge the ever-expanding list of genes associated with dementia since the study's initiation in 2017. Our targeted gene panel was updated at the end of the study, and we advise using this updated panel for genetic testing (eTable 6). Finally, it is a limitation that the genetic ancestry from majority of patients in this study was European. Therefore, the performance of the criteria may differ in populations with a different mix of genetic ancestry groups.

To date, only one other study searched for genetic causes of dementia in a large number of samples.^2^ This study by Koriath et al.^14^ investigated a substantial referral-based dementia cohort encompassing AD, FTD, and prion diseases. They found deleterious variants in 12.6% of all participants using a targeted dementia gene panel consisting of 17 genes. In our single-site clinical cohort within a specialized memory clinic, the prevalence of PGVs was lower (3.3%). This is a seemingly large difference, but the patient populations were different. Koriath et al.^2^ included 24.4% FTD patients (compared to 5% in ours) and 9.2% had a prion disease (compared with 0.2% in our study). The number of PGV carriers in these 2 groups were high (>20%), increasing the total percentage of patients with a PGV. In addition, the number of patients without neurodegenerative disease was higher in our study (32%) compared with Koriath et al. (14%).^2^ If we match the percentage of diseases (and controls) of Koriath et al.^2^ to our population, the percentage of PGV carriers in Koriath et al. would be between 4% and 5%, which is only slightly higher than our estimate.^2^

The eligibility criteria from previous studies for genetic testing in dementia patients combine clinical phenotype, an early onset, or a strong family history of dementia, resulting in diagrams of varying complexity.^1,2,7,14,26-29^ The diagrams are often developed for separate diseases, such as AD,^16,28^ FTD, HTT, and prion diseases,^2^ or for criteria based on well-defined patient cohorts,^2^ or for cases of unclear etiology.^30^ Our new criteria resemble most the procedure for eligibility presented by Koriath et al.^14^ We used age at presentation instead of age at onset and put less emphasis on family history. We used the age at presentation instead of age at onset because it is easier for the clinician and is not influenced by recall bias. The age cut-offs implemented are also much less strict.^31^ Less emphasis on an early age at symptom onset is necessary because we found that 53% of patients with a PGV were older than 60 at time of presentation, replicating previous findings.^2,32^ For family history, the most used criteria are the Goldman criteria for genetic testing for patients with FTD^29^ and AD.^33^ In practice, the etiology of disease may not be clear in family members, and family history may be limited or even absent,^14^ therefore we simplified family history to only first degree relatives. Still, if present, clinicians should consider strong multigenerational family history of cognitive complains, ALS, or late-onset psychiatry because it increases chances to find a PGV.^2,7,29^ We advise to refer to a geneticist when there is a strong family history of dementia, and/or unexplained psychiatric diagnosis in the family.

Worldwide, patients with dementia that carry a PGV are not identified because of multiple factors, including practical and financial issues.^34^ In some countries, genetic testing facilities may be inaccessible or entirely absent. In other countries, accessibility may be limited due to out-of-pocket expenses (e.g., in the United States). In most European countries, however, genetic testing is available and the costs of genetic testing are covered by insurances. Here, the public has a right to expect that the obligatory insurance premiums will not be used to pay for ineffective or unnecessary tests. Therefore, only the request of a patient to be tested is not enough to offer genetic testing. The criteria were designed and validated in the Dutch health care system, and hence, it is specifically relevant for countries and clinical practices in which there are no practical or financial issues related to genetic testing.^34^ In such an environment, the new criteria can provide a balance in offering genetic testing to all symptomatic patients that could carry a pathogenic variant while avoiding “over testing.”

For organizing care, it is essential to have an estimated number of patients eligible for genetic testing. We estimate that approximately 1 in 5 patients younger than 70 years at presentation, regardless of their diagnosis, are eligible and want to be tested and that 38% of patients tested will need specialized counseling. Between the ages of 70 and 75 years, approximately 1 in 20 patients is eligible for genetic testing, and (nearly) no patients aged 75 years and older. These estimates may be lower in less specialized memory clinics, in health care systems with more self-payment and varying legal protections in different countries.

In conclusion, routine assessment of eligibility for genetic testing in all patients with dementia will improve the identification of patients with familial dementia. Implementation requires minor change from “standard” clinical practice, and, with the new eligibility criteria, clinicians can offer genetic testing to the right patients, leading to more personalized management options for patients with dementia.

## Glossary

ACMG: American College of Medical Genetics and Genomics
AD: Alzheimer disease
ALS: amyotrophic lateral sclerosis
FTD: frontotemporal dementia
IQR: interquartile range
NGS: next-generation sequencing
PGV: pathogenic genetic variant
VUS: variants of unknown significance
